# The Advantage of Standing Up to Fight and the Evolution of Habitual Bipedalism in Hominins

**DOI:** 10.1371/journal.pone.0019630

**Published:** 2011-05-18

**Authors:** David R. Carrier

**Affiliations:** Department of Biology, University of Utah, Salt Lake City, Utah, United States of America; University of Wisconsin, United States of America

## Abstract

**Background:**

Many quadrupedal species stand bipedally on their hindlimbs to fight. This posture may provide a performance advantage by allowing the forelimbs to strike an opponent with the range of motion that is intrinsic to high-speed running, jumping, rapid braking and turning; the range of motion over which peak force and power can be produced.

**Methodology/Principal Findings:**

To test the hypothesis that bipedal (i.e., orthograde) posture provides a performance advantage when striking with the forelimbs, I measured the force and energy produced when human subjects struck from “quadrupedal” (i.e., pronograde) and bipedal postures. Downward and upward directed striking energy was measured with a custom designed pendulum transducer. Side and forward strikes were measured with a punching bag instrumented with an accelerometer. When subjects struck downward from a bipedal posture the work was 43.70±12.59% (mean ± S.E.) greater than when they struck from a quadrupedal posture. Similarly, 47.49±17.95% more work was produced when subjects struck upward from a bipedal stance compared to a quadrupedal stance. Importantly, subjects did 229.69±44.19% more work in downward than upward directed strikes. During side and forward strikes the force impulses were 30.12±3.68 and 43.04±9.00% greater from a bipedal posture than a quadrupedal posture, respectively.

**Conclusions/Significance:**

These results indicate that bipedal posture does provide a performance advantage for striking with the forelimbs. The mating systems of great apes are characterized by intense male-male competition in which conflict is resolved through force or the threat of force. Great apes often fight from bipedal posture, striking with both the fore- and hindlimbs. These observations, plus the findings of this study, suggest that sexual selection contributed to the evolution of habitual bipedalism in hominins.

## Introduction

Although bipedal locomotion is rare among mammals, many species stand bipedally on their hindlimbs when they fight. Fighting from a bipedal posture is commonly observed in anteaters, felids including domestic cats, lions and tigers; canids including foxes, wolves and domestic dogs; bears; wolverines; horses; and many species of rodents, lagomorphs and primates, including great apes. Why is this behavior so common among species that normally stand, walk and run on four legs? The simplest answer is that bipedal posture allows a quadruped to fight with its forelimbs. Among extant tetrapods, mammals are remarkable in the mobility of their forelimbs and their ability to grab, hold and manipulate objects with their forelimbs [1], [2]. Given this mobility and dexterity, it is not surprising that many mammals fight with their forelimbs. Nonetheless, bipedal posture may also bestow specific advantages for fighting with the forelimbs that emerge from the mechanics of quadrupedal locomotion and the contractile physiology of striated muscle.

Terrestrial vertebrates have evolved to do work against gravity during locomotion. This requires that the mobility and strength of limbs be oriented towards the substrate. Bipedal posture reorients the trunk from pronograde to orthograde, allowing quadrupeds to defend themselves and strike and manipulate an opponent with their forelimbs over the locomotor range of motion; the range of motion that can presumably produce the most force and power. Consider a galloping thoroughbred horse. At full speed, each forelimb is in contact with the ground for much less than a tenth of a second and, during that brief period, it applies a peak ground force of more than 2.5 times body weight [3]. Thus, bipedal posture repositions the axis of the body so that the locomotor range of motion of the forelimbs can be directed at an opponent, allowing quadrupeds to strike, grapple and defend themselves with their forelimbs' greatest capacity to do work.

The force-velocity relationship of striated muscle may also influence body posture during aggressive encounters. Bipedal posture allows quadrupeds to strike downward rather than upward on an opponent. Striking downward may increase the power of the limb because limb retractor muscles have a greater capacity for positive work than limb protractor muscles. In quadrupeds, retractor muscles are primarily responsible for the positive work associated with accelerating the body whereas protractor muscles apply force during braking and are therefore responsible primarily for negative work [4], [5]. Muscle fibers produce more force during active lengthening (i.e., eccentric activity) that is required during braking than during active shortening (i.e., concentric activity) that is required for acceleration [6]. Thus, if quadrupeds need to slow down as rapidly as they need to speed up, one would expect the protractor muscles to have smaller physiological cross-sectional area than the retractor muscles. This appears to be true; protractor muscles are substantially smaller than retractor muscles in a variety of species [7]–[9]. Because striking downward requires concentric activity from the retractor muscles of the forelimbs whereas striking upward requires concentric activity from the smaller protractor muscles, quadrupeds may be able to do more work on an opponent when they strike downward.

These observations lead to two predictions. First, mammals will strike with greater force and power from bipedal posture than from quadrupedal posture. Second, mammals are expected to exhibit greater force and power when they strike down than when they strike up with their forelimbs. These predictions would be best tested in habitual quadrupeds. Practical limitations, however, make such an experiment relatively difficult. Thus, to test these predictions, I quantified striking performance of human subjects (1) in bipedal (orthograde) posture and in simulated quadrupedal (pronograde) posture, and (2) when striking downward versus upward. Although humans are highly derived striding bipeds, our forelimbs do play a critical locomotor role in climbing. During climbing, the range of motion at the shoulder is largely similar to that used during terrestrial quadrupedalism and the muscles at the shoulder responsible for positive work during climbing are the retractors. Additionally, although quadrupedal posture is not the preferred fighting posture of humans, it does occur during grappling and ground fighting. Thus, human subjects do provide a valid test of the predictions.

## Results

### Striking performance in quadrupedal versus bipedal posture

**Figure 1:**
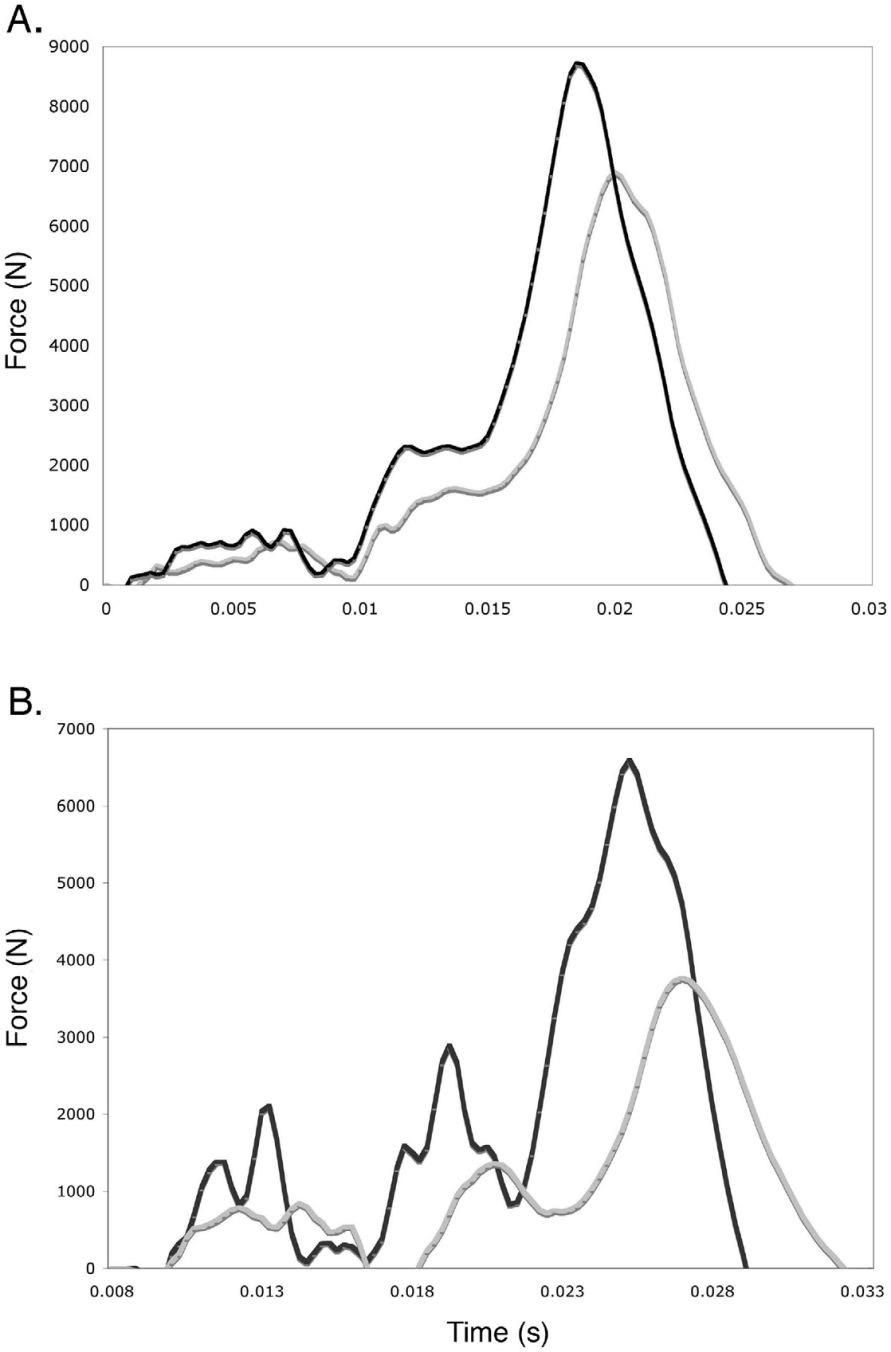
Sample recordings of straight (i.e., forward) punches (A) and side strikes (B) from quadrupedal (gray lines) and bipedal (black lines) posture.

For all four types of strikes (downward, upward, side and forward), performance by the subjects was greater from bipedal than from quadrupedal posture. When striking downward and upward, subjects did 44 and 47% more work respectively when they performed from bipedal than from “quadrupedal” posture (Table 1). When the subjects punched the transducer with a forward strike, peak forces averaged 49% greater and force impulses averaged 30% greater from bipedal than from quadrupedal posture (Fig. 1A, Tables 2 and 3). When striking sideways, peak forces were on average 64% greater and force impulses averaged 43% greater from bipedal than quadrupedal posture (Fig. 1B, Tables 2 and 3).

**Table 1:** Mean values and standard errors of work (J) done during striking downward and upward from “quadrupedal” and bipedal posture.

Strike	N	Quadrupedal	Bipedal	P-value^1^	% difference
Downward	12	23.47±2.62	32.33±3.69	0.0018	43.71±12.59
Upward	9	8.90±1.37	11.98±1.49	0.014	47.49±17.95

P-value^1^ - Student t-test.

**Table 2:** Mean values and standard errors of maximum force (N) delivered during side and forward strikes from “quadrupedal” and bipedal posture.

Strike	N	Quadrupedal	Bipedal	P-value^1^	% difference
Side	12	3,196±163	5,193±439	0.0002	63.85±12.73
Forward	9	3,968±399	5,785±453	<0.0001	48.72±7.30

P-value^1^ - Student t-test.

**Table 3:** Mean values and standard errors of force impulse (Ns) delivered during side and forward strikes from “quadrupedal” and bipedal posture.

Strike	N	Quadrupedal	Bipedal	P-value^1^	% difference
Side	12	22.91±0.69	32.82±2.33	0.0003	43.04±9.00
Forward	9	34.16±2.16	44.17±2.41	<0.0001	30.12±3.68

P-value^1^ - Student t-test.

### Performance in downward versus upward directed strikes

The work done in maximal effort strikes was 2.3-fold greater in downward than upward strikes when the subjects performed from both bipedal and quadrupedal posture (Table 4). This large difference was readily apparent to the subjects; most commented on their limited ability to strike upward compared to downward.

**Table 4:** Mean values and standard errors of work (J) done from bipedal and quadrupedal posture when striking downward and upward.

Posture	N	Down	Up	P-value^1^	% difference
Bipedal	9	36.12±3.77	11.67±1.22	0.00006	229.7±44.19
Quadrupedal	9	25.62±2.64	8.90±1.37	0.0001	228.0±46.62

P-value^1^ - Student t-test.

## Discussion

### The advantage of fighting from bipedal posture

The results of this study indicate that humans are capable of striking with 40–50% higher force and energy from bipedal than quadrupedal posture and can impart more than 200% greater energy when striking downward than upward. The increase in work done in downward and upward strikes when subjects switched from quadrupedal to bipedal posture likely reflects the difference in the range of motion of the arm in these two postures. When subjects struck vertically from a bipedal posture, they used approximately the full range of motion of the arm. In contrast, when they struck vertically from a quadrupedal posture, the motion was restricted to relatively protracted angles, limiting the range of motion by roughly half of the full range. Additionally, although kinematics were not quantified, subjects tended to raise their arm into extreme protraction in preparation for the quadrupedal strikes. Power production is likely to be limited by the length-tension relationship of the extrinsic shoulder muscles at these joint angles.

The greater performance in side and forward directed strikes from bipedal posture is partially a function of a transfer of energy from the legs and trunk that bipedalism makes possible. Although the contribution of legs and trunk to the work of side and forward strikes was not addressed in this study, energy transfer from the legs and trunk is generally recognized to be important in fighting. However, because one forelimb remains in contact with the ground, quadrupedal posture largely eliminates significant contribution from the trunk and legs. The greater performance in side and forward strikes from bipedal posture is also likely a consequence of the relative strength of the different shoulder muscles producing these two movements. From a bipedal posture, both side and forward strikes require (1) adduction of the humerus, produced by the pectoralis major and anterior deltoid muscles, and (2) anteversion of the arm on the trunk produced by the serratus anterior muscle. From quadrupedal posture, however, side and forward strikes require lateral “elevation” of the arm in which the humerus is brought closer to the head. Elevation of the arm is produced primarily by the middle deltoid muscle. In humans, the combined physiological cross-sectional area of the pectoralis major and anterior deltoid muscles is approximately 130% larger than that of the middle deltoid muscle [10], [11]. Additionally, side and forward striking from a quadrupedal posture eliminates the contribution from the powerful serratus anterior muscle that likely occurs during bipedal horizontal strikes.

The more than two-fold greater work done in downward than in upward directed strikes is consistent with the greater strength of the retractor than the protractor muscles of the forelimb. As mentioned above, retractors of the forelimb tend to have greater physiological cross-sectional area than do the protractors in mammals [8], [9]. This is also true of humans, in which the latissimus dorsi, the sternocostal part of the pectoralis major, the teres major and the long head of the triceps all act as retractors of arm. In comparison, only the anterior and middle portions of the deltoid and the clavicular part of the pectoralis major muscle have a capacity to protract the arm. Nevertheless, for the biomechanical reasons described above, a greater capacity to strike downward than upward is likely to be true for most species of mammals, including the quadrupedal ancestors of hominins.

The imbalance of strength in retractor versus protractor muscles of the limbs of quadrupeds, such as hares and dogs, is almost certainly a consequence of the mechanics of running on four legs and the force velocity relationship of skeletal muscle, as explained above. Given that habitual bipedalism evolved over 4 million years ago in hominins, why have humans retained this imbalance in muscular strength in our forelimbs? One possibility is the role that forelimb retractors play in the production of positive and negative work during climbing. Another factor that may have been important is that our ancestors evolved overhand motor behaviors associated with aggression, long before the evolution of habitual bipedalism. Chimpanzees, bonoboos and gorillas all strike opponents with overhand motions of the forelimb. Chimpanzees also throw with an overhand motion of the forelimb. Overhand striking and throwing are more common in apes than underhand versions of these same behaviors presumably because of the greater capacity of their forelimbs to do positive work during retraction than during protraction. We inherited overhand motor control of these behaviors from our quadrupedal ancestors. The necessity of high power production during striking and throwing may be why humans retained a greater capacity for power production during retraction rather than protraction of the forelimb.

The fact that humans are habitual bipeds reduces the relevance of humans as a model organism for this study. Obviously, the biomechanical predictions of this study would be better tested in a species that walks and runs quadrupedally. Collecting similar data from chimpanzees or bonoboos may be possible, but will be difficult for a variety of reasons and confounded by questions of motivation and training. Nevertheless, a study similar to this one in another species of great ape would be worthwhile.

In summary, humans are capable of striking with greater force and energy from bipedal than quadrupedal posture and can impart much more energy when striking downward than upward. The magnitude of the greater energy imparted in downward directed strikes suggests that the most important reason quadrupeds stand bipedally to fight is that it allows them to strike downward on an opponent.

### A functional basis for the attractiveness of tall males

All else being equal, the much greater energy that can be delivered in downward than in upward directed strikes provides a tall individual with a performance advantage over a shorter opponent. This height dependent advantage may be the basis of the observed female preference for tall men. Several studies have found that women are more attracted to tall than short men [12]–[14]. Tall men receive more responses to dating advertisements [15] and women report dating tall men more often than short men [16]. In the latter study, men one standard deviation above the mean height had twice as many dates as did men one standard deviation below the mean. Tall men also have more attractive partners [17], report greater relationship satisfaction and have lower levels of cognitive or behavioral jealousy than short men [18], [19]. These differences in male attractiveness and relationship confidence appear to give taller men a fitness advantage. Tall men have a greater number of children than shorter men [20]–[22]. Furthermore, given that stature is highly hertiable [23], females who mate with tall men are more likely to have tall sons, who in turn would be preferred by females.

The greater attractiveness and reproductive success of taller males is generally assumed to be due to stature serving as an indicator of good genes. Height is correlated with cognitive abilities and is positively associated with a number of metrics of social and financial success [24], [25]. Height has also been found to be correlated with physical health and with morphological symmetry [26], [27]. Yet, if the greater attractiveness and reproductive success of taller men were solely a function of the correlation with somewhat greater intelligence, health and social success, we could expect taller women to be more attractive to men and to have greater reproductive success for the same reasons. In western societies, this is not the case. Women who are short or of average height are perceived as more attractive by men [16], [28], have lower levels of jealousy [18], and have greater reproductive success [29] than tall women. Thus, the presence of “good genes” is unlikely to account fully for the greater attractiveness and reproductive success of taller males.

A performance advantage in male-male competition could also be part of the explanation for the greater attractiveness of taller males. Although larger size (i.e., body mass) provides an advantage during physical competition, the results of this study suggest that greater height, by itself, is associated with an enhanced capacity to strike downward on an opponent. Short individuals have to strike upward to hit a tall person in the head, but tall fighters swing downward to hit the most vulnerable targets of a shorter opponent. Consistent with this, is the observation that tall men are perceived to be more dominant and assertive than shorter individuals [30]. Thus, early in hominin evolution, an enhanced capacity to strike downward on an opponent may have given tall males a greater capacity to compete for mates and to defend their resources and offspring. If this were true, females who chose to mate with tall males would have had greater fitness.

### Fighting and the evolution of bipedal posture in hominins

The primary weapons of most primates are their jaws and large canine teeth. Darwin [31] recognized the association of an increased use of the forelimbs in fighting and a reduction in size of their canine teeth in apes. In doing so, he associated habitual bipedal posture with fighting with the forelimbs. More recently, Livingstone [32] used observations of aggressive bipedal chest-thumping, charging and fighting in gorillas to argue that aggression was fundamental to the evolution of hominid bipedalism. Wescott [33] suggested the evolution of bipedalism in hominins was associated with aggressive displays in which bipedal posture made the individual appear larger and more threatening and Guthrie [34] echoed this suggestion. Kortlandt [35] suggested that early hominins adopted an upright posture and gait to be able to wield thorn branches as a defense against their predators. Most recently, Jablonski and Chaplin [36] have suggested that the bipedal behaviors that were critical to the differentiation of the Hominidae were those involved in the control of intragroup aggression and conflict. The most important behaviors in this context, they argue, were bipedal displays, bipedal charges and bipedal mock fights. Indeed, a recent behavioral study in captive chimpanzees found that both postural and locomotor bipedalism are strongly associated with aggressive behavior in males but not in females [37]. Thus, the idea that aggression played an important role in the evolution of habitual bipedalism has been proposed many times. Although the aggressive ape hypothesis appears not to be taken seriously by many scholars of human evolution, there are several reasons why it should be.

The possibility that specialization for aggression has influenced the evolution of great apes warrants consideration because great apes are a relatively aggressive group of mammals. Intense male–male aggression is prevalent among males of all species of extant Hominidae [38], [39]. Fighting behavior of apes has been most thoroughly described in chimpanzees [40]–[43]. Coalitional killings have been reported from 8 out of 10 chimpanzee study populations [44], [45], and the deaths can represent a significant proportion of the population [44], [46]. Male–male aggression among bonobos appears similar in many ways to that of chimpanzees, but of a lower intensity. The vast majority of aggressive interactions in bonobos are between adult males [47]–[49]. Although most aggressive interactions do not involve physical contact, when aggression escalates bonobos bite, hit, kick, slap, grab, drag, shove aside and pin down [49]. Mature male orangutans are reported to be totally intolerant of each other [50]. Of two observed encounters between adult males in the presence of adult females, both “entailed considerable physical violence” [50]. One of these fights lasted for over half an hour and involved “bouts of grappling in the canopy and on the ground with the males biting each other's hands, head and shoulders.” In gorillas, male–male aggression during intergroup encounters is common. Harcourt [51] reported that violent displays occur during 80% of these encounters and fights between males occur during 50% of the encounters. Gorillas also display twice the prevalence of cranial trauma (11%) as chimpanzees and this trauma is thought to be primarily associated with male-male aggression [52]. Among extinct species, the size sexual dimorphism of early hominins, such as *Australopithecus*, is suggestive of polygynous mating systems and significant intermale physical competition [53]–[55]. Characters such as pronounced forelimb strength, robust distal limbs, short stature, wide hips, robust head and neck, and habitual bipedalism gave australopiths a body configuration that is consistent with specialization for fighting with the forelimbs [39], [56]. These traits plus a high level of sexual dimorphism in upper body and forelimb size suggest that australopiths were specialized for male–male aggression. *Homo sapiens* is also a relatively violent species and much of the aggressive behavior observed in modern humans appears to be a consequence of male–male competition [57]–[67]. Thus, the mating systems of great apes, including humans, are characterized by male–male competition [38], [54] that subjects males to intense sexual selection on fighting performance [67].

A second reason to suggest that aggression may have influenced the evolution of habitual bipedalism in hominins is that fighting is one of the few behaviors in which all species of great apes routinely adopt bipedal posture [32], [37], [38], [42], [48]. In this case, also, the most complete observations come from field studies of chimpanzees [37], [42]. Coalitional attacks by groups of male chimpanzees often begin by grappling and pulling the victim to the ground, in some cases from out of a tree the victim had attempted to flee into [42]. The victim is held, pinned to the ground by one or two members of the group while other members attack by biting, hitting with fists, and kicking and stomping with the hindlegs. The victims are often dragged for distances on the ground, lifted and slammed back to the ground, and attempts are often made to break arms and legs by twisting. Many of these fighting techniques are dependent on bipedal posture. Chimpanzees also stand bipedally to use weapons such as rocks and clubs [35], [38], [41], [42].

Bipedal posture also plays a critical role in the threat displays of great apes. Threat displays provide important clues to the weapons used in fighting. Game theory modeling of aggressive encounters suggests that threat displays, when they exist, usually provide an honest indication of one's fighting ability [68]–[72]. All known examples of threat displays illustrate weaponry and fighting technique. Importantly, it is usually the first step in a species' fighting technique that is used to threaten [71], [73]. In chimpanzees and bonobos, the most dramatic threat is the charging display. This display includes running along the ground, often bipedally; dragging or flailing branches; throwing rocks or other loose material; slapping the ground with the hands and stomping with feet, or both alternately; and leaping up to hit and stomp on a tree [37], [42], [47], [74]. These displays emphasize strength and agility in a bipedal stance and the power with which an individual can hit with his forelimbs and stomp with his hindlimbs. Terrestrial bipedal threat displays appear to be basal to the *Gorilla*, *Pan*, *Homo* clade and are indicative of a fighting strategy in which the limbs are important weapons used to punch, slap, kick, stomp and twist.

The results of this study are also consistent with the evolution of habitual bipedalism being associated with male-male competition. Standing up on the hindlimbs allows a quadruped to strike and manipulate its opponent with the forelimbs over the same range of motion used during locomotion. Humans are capable of striking with substantially higher force and energy from bipedal than quadrupedal posture. Bipedality also facilitates striking downward which can impart more than 200% more energy than is possible when striking upward. Downward strikes are more powerful because the retractor muscles of the forelimb are much stronger than the protractor muscles; a difference that emerges from the locomotor division of labor of protractor and retractor muscles and the force-velocity relationship of skeletal muscle.

In conclusion, several observations suggest that selection for improved fighting performance may have played an important role in the evolution of habitual bipedalism in hominins. Great apes are a relatively aggressive group of mammals. Their mating systems are characterized by intense male–male competition in which conflict is resolved through force and the threat of force. We can be confident that bipedal posture is important in these aggressive encounters because great apes often fight, and threaten to fight, from a bipedal posture. Furthermore, the results of this study indicate that in addition to freeing the arms to strike and grapple with an opponent, bipedal posture allows a significant increase in the force and power of forelimb strikes. Many hypotheses have been proposed regarding the selective advantages of bipedal posture in early hominins: locomotor economy [75], [76], locomotor stamina and persistence hunting [77], aquatic foraging/wading [78], thermoregulation in a hot environment [79], carrying [80], [81], carrying infants [82]–[84], reaching for food [85], male provisioning of females [86], arboreal foraging in the distal branches of trees [87], freeing of hands [31], defense against predators [35], [88], and aggressive encounters [36], [37], [56]. Walter's [88] hypothesis of passive defense against predators is particularly interesting. He offers compelling evidence that predators such as leopards and tigers preferentially attack humans when they are seated, crouched or lying rather than when they are standing. Reluctance by predators to attack an erect human would make sense if great apes are most dangerous when they stand bipedally. Although some of the many hypotheses for the evolution of habitual bipedalism appear more plausible than others, given current knowledge, it is likely that more than one selective factor was involved. The results of this study and the behavior of great apes suggest that sexual selection, associated with male-male competition, contributed to the evolution of habitual bipedalism in hominins.

## Methods

### Ethics Statement

Subjects gave informed written consent and all procedures were approved by the University of Utah Internal Review Board.

### Subjects and protocol

**Figure 2:**
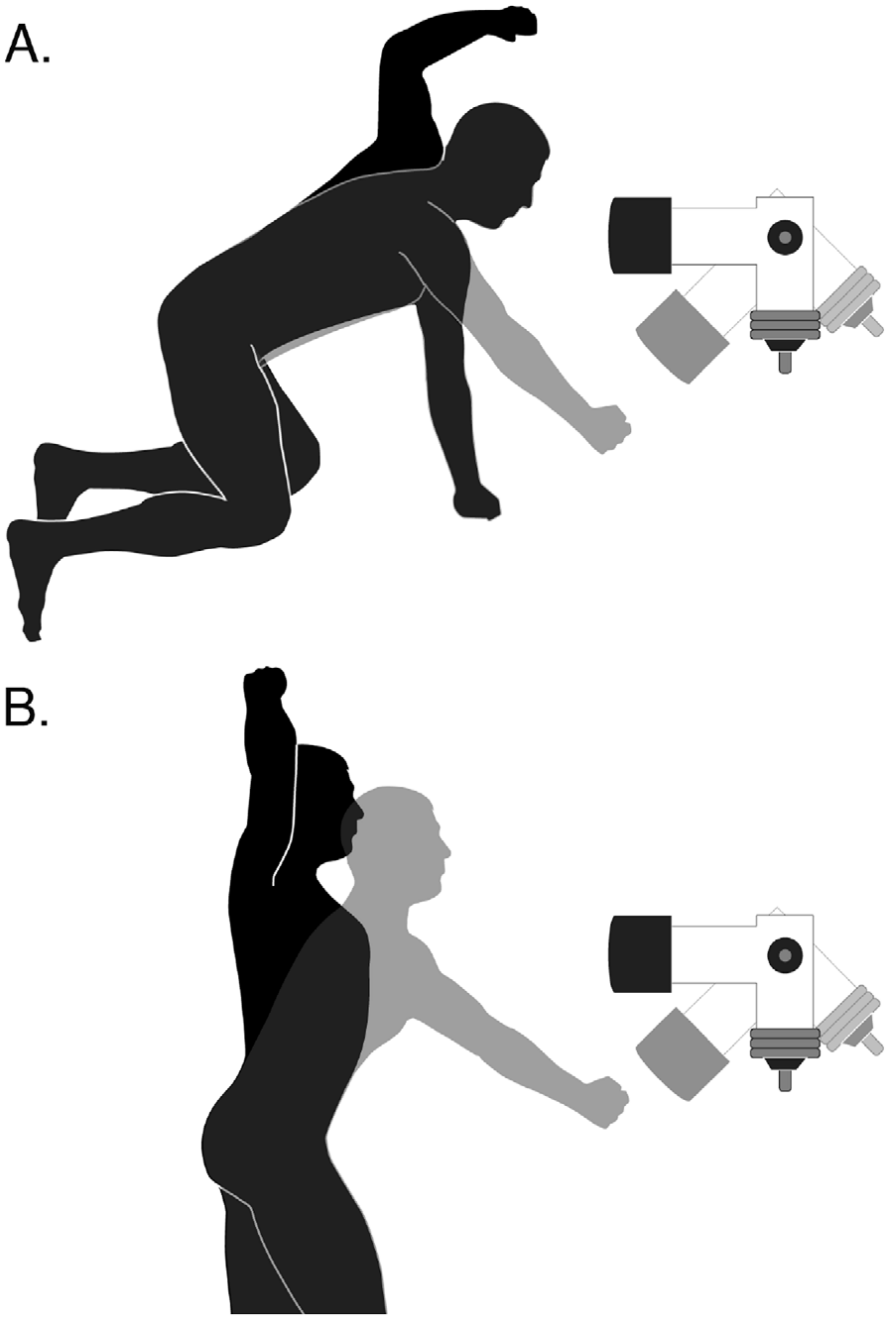
Illustrations of the pendulum transducer used to measure the energy imparted during maximum effort vertically directed strikes from (A) quadrupedal and (B) bipedal posture. The starting posture of the subject and position of the transducer are shown in black. Body posture and the swing of the transducer after the strike are illustrated with grey.

The work done on, or the force delivered to, the transducers was measured during maximal effort strikes by human subjects in quadrupedal and bipedal postures (Fig. 2). In the “quadrupedal” posture, subjects supported themselves on their knees and one arm (i.e., tripedally), such that their trunk was oriented horizontally (pronograde), while they struck with the other arm. In the bipedal posture, subjects stood on both legs, with their trunks oriented vertically (orthorgrade). All subjects (15 males, body mass 78.8±7.8 kg; age 34.5±10.3; means ± S.D.) were healthy and experienced fighters having received at least 6 months of training in boxing or martial arts prior to the study. Subjects chose the arm, right or left, they used to strike the transducer. Subjects gave informed consent and all procedures were approved by the University of Utah Internal Review Board.

Experienced fighters were used as subjects to minimize the risk of injury and to reduce biases in performance among the different types of strikes that were studied. Because humans typically have more experience in overhand rather than underhand power motions of the arm, problems with motor control could limit performance when striking upward in untrained subjects. The experienced subjects, used in this study, hopefully minimized this potential bias.

Striking performance from quadrupedal posture was compared to that from bipedal posture for four types of strikes: downward, upward, side and forward strikes. Subjects warmed up and became familiar with the transducers by striking with sub-maximal effort. Once the subject felt warmed up and confident about the tasks, they were asked to strike the transducer as hard as possible three times in a row, with a rest of approximately 60 seconds between strikes. To avoid an artifact due to muscular fatigue, half of the subjects began their recording sessions striking from quadrupedal posture and the other half started from bipedal posture.

### Vertically oriented strikes

**Figure 3:**
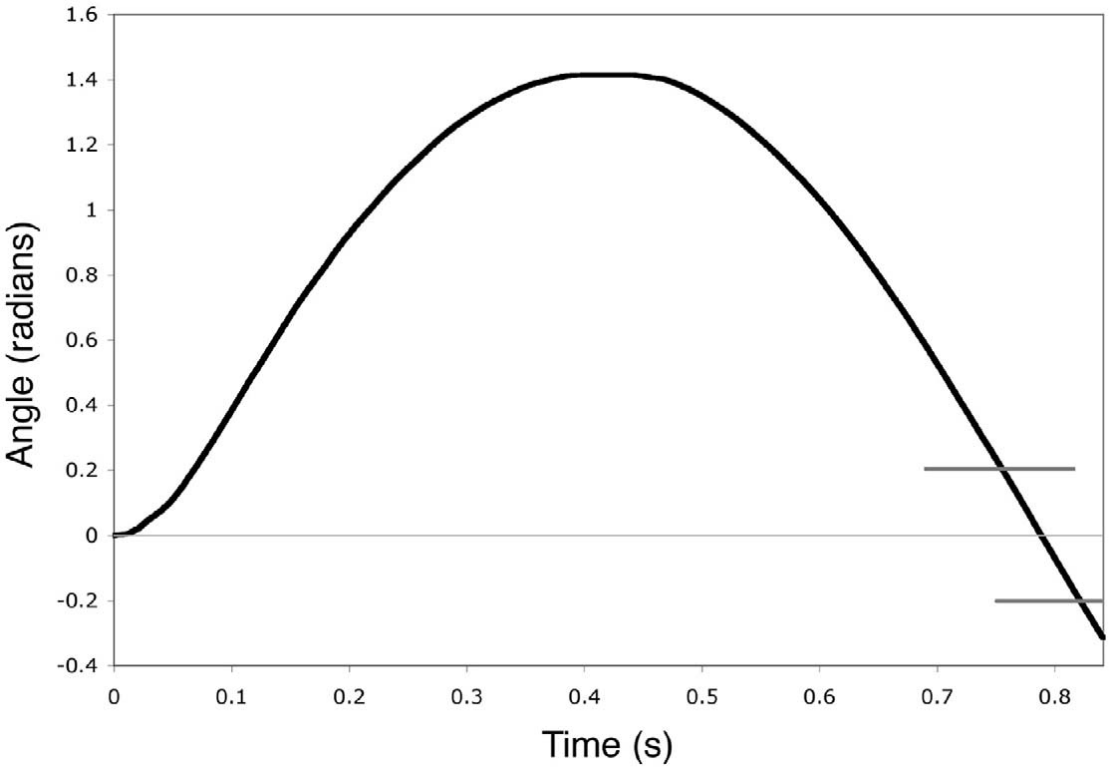
Sample recording of the energy delivered in maximum effort downward and upward directed strikes. Contact with the transducer was made at time zero. The force of the strike caused the pendulum to swing upward. Maximum potential energy occurred at the peak of the curve. Maximum kinetic energy occurred as the pendulum swung back to the bottom of its arch at 0 radians. To calculate the maximum kinetic energy of the pendulum, the angular velocity (ω) of the pendulum was measured as it swung through the bottom of its arc, over an angular displacement of +0.20 to −0.20 radians, indicated by the horizontal grey lines.

To determine the work done in maximal effort downward and upward directed strikes, subjects struck a stationary pendulum and I calculated the change in its kinetic energy. The pendulum was constructed from a laminated block of plywood and was attached to an axle that turned in bearings mounted in a support frame (Fig. 2). To increase inertia, dumbbell weights were attached to the bottom of the block, directly below the axis of rotation. Subjects struck an arm of the block that extended horizontally from the axis of rotation. The pendulum had a mass (M) of 30.80 kg. The distance (D) from the center of mass to the axis of rotation was 0.143 m. The pendulum's period (T) was 1.28 s. Its rotational inertia (I) was calculated using the formula I = (T^2^MgD)/4π^2^, and was found to be 1.79 Nm^2^. Angular displacement of the pendulum was monitored with a Wheatstone bridge of which one arm was a linearly variable resistor mounted to the frame such that the rotor of the variable resistor turned with the axle of the pendulum. Digital data were collected on a computer with a sampling rate 4,000 Hz. I measured the angular velocity (ω) of the pendulum as it swung back to the bottom of its arch, over an angular displacement of +0.20 to −0.20 radians from the vertical; i.e., the portion of the swing in which kinetic energy and velocity are greatest (Fig. 3). The change in kinetic energy, and therefore the work done in the striking the pendulum, was calculated from the equation: K_E_ = 0.5Iω^2^.

The height of the platform on which each subject stood was adjusted by adding 12 cm wooden pallets and/or 2 cm sheets of plywood until the subject's shoulder joint (i.e., glenoid) was level with the target of the transducer in both bipedal and quadrupedal postures. For the comparison of quadrupedal and bipedal posture, 12 subjects (body mass 78.5±8.6 kg; age 33.7±9.9) participated in the measurement of downward directed strikes (i.e., overhead, hammer fist) and 9 of these subjects (body mass 79.6±9.1 kg; age 31.7±9.7) also participated in the measurement of upward directed strikes (i.e., uppercut, thumb directed hammer fist). Thus, nine subjects (body mass 79.6±9.1 kg; age 31.7±9.7) participated in the comparison of downward versus upward directed strikes.

### Horizontally oriented strikes

To measure the force and force impulse of horizontally directed strikes, subjects struck a punching bag close to its center of mass. I measured the acceleration of the bag and then multiplied the instantaneous acceleration of the bag by the bag's mass to get the instantaneous force. I measured acceleration of the bag with an Endevco model 7290A-10 Microtron accelerometer (San Juan Capistrano, CA, USA) attached to the outside of the bag lateral to its center of mass. The accelerometer had a working range of −18 to +19 g. Digital data were collected on a computer with a sampling rate 4,000 Hz. The punching bag had a mass of 45.45 kg and was suspended from the ceiling of the laboratory with chains 2.0 m long. For the comparison of quadrupedal and bipedal posture, 12 subjects (body mass 80.3±8.1 kg; age 36.8±10.4) participated in the measurement of side directed strikes (i.e., side strike, hammer fist) and 9 of these subjects (body mass 80.7±9.1 kg; age 34.4±11.8) also participated in the measurement of forward directed strikes (i.e., forward punch).

### Analyses

For each of the tests, subjects struck the transducer 3 times with maximal effort. I tested for differences in the average work or force of the three strikes from quadrupedal and bipedal postures in downward, upward, side and forward directed strikes. I also tested for differences in the average work of maximal effort for the upward versus the downward directed strikes in both quadrupedal and bipedal posture. Tests of difference were done using paired student t-Tests. I used a one-sided test for significance given the hypotheses that bipedal posture would be more effective than quadrupedal posture and that striking downward would result in more powerful recordings than upward strikes. I assumed the results were significantly different when the *p*-value was less than 0.05.
